# Supplementary material for: The genome sequence of the spotted Meliscaeva, Meliscaeva auricollis (Meigen, 1822)
Source: Wellcome Open Res. 2024 Feb 19;9:82. [Version 1] doi: 10.12688/wellcomeopenres.20843.1 (PMC11316168; doi:10.12688/wellcomeopenres.20843.1)
Supplement: Peer review history

# Reviewer report: Reviewer response for version 1

**DOI:** 10.21956/wellcomeopenres.23062.r89138

**Published:** 2024-08-09

**Authors:** Luis Diambra

**Affiliations:** 1 Univeridad Nacional de La Plata, La Plata, Argentina

This manuscript from Falk and Woodcock reports the genome of *Meliscaeva auricollis*. The raw genomic data, extracted from an individual female, was generated by Pacific Biosciences single-molecule HiFi. These reads were assembled using Hifiasm (35-fold coverage) and scaffolded to Hi-C data using YaHS. The assembly was scaffolded into 5 chromosomal pseudomolecules. Manual curation was performed using HiGlass. The authors provide the assembly statistics obtained by using BlobToolKit and BUSCO. The authors do not perform the genome annotation, despite the RNA sequencing. All these analyses are clearly explained in the protocols of the Tree of Life, which contain enough information to allow replication by other researchers. However, the authors do not mention the software/pipeline for the decontamination step (endosymbionts, etc). They only mention the Howe 2021 paper. Could the authors give more details about that?

Are sufficient details of methods and materials provided to allow replication by others?

Yes

Is the rationale for creating the dataset(s) clearly described?

Yes

Are the datasets clearly presented in a useable and accessible format?

Yes

Are the protocols appropriate and is the work technically sound?

Yes

Reviewer Expertise:

Bioinformatics

I confirm that I have read this submission and believe that I have an appropriate level of expertise to confirm that it is of an acceptable scientific standard.

# Reviewer report: Reviewer response for version 1

**DOI:** 10.21956/wellcomeopenres.23062.r88077

**Published:** 2024-08-05

**Authors:** Adrián Casanova Chiclana

**Affiliations:** 1 Department of Zoology, Genetics and Physical Anthropology, Universidade de Santiago de Compostela, Lugo-Terra Campus, Galicia, Spain

To my view,  this research is clear and well-conducted. I have made some suggestions across sections.

**Keywords**

 I would recommend using "chromosome assembly" instead of "chromosomal". I consider more informative for the readers the first one.

**Background**

 It might be adequate to add in the first paragraph more information regarding to distribution range, conservation status and its role in ecosystems in a brief way. I would add in one sentence (before the sentence about its higher frequency in England), the distribution range of this species and its conservation status. You can see, for instance in the IUCN RedList: https://www.iucnredlist.org/species/149167300/149167303

 At the end of the first paragraph you could add one more specific sentence about its role as pollinator, predator of aphids, etcetera as you consider. You could add information from the Global Biotic Interactions (GloBI) database [1], for instance: https://www.globalbioticinteractions.org/?interactionType=interactsWith&targetTaxon=Meliscaeva%20auricollis

**Genome sequence report**

 If well the percentage of uncalled nucleotides (i.e., "N") is very low (0.0%; Figure 2) perhaps it could be included somewhere the number of them. I think that 32,837 uncalled nucleotides. Add if you agree, it does not matter really.

**Methods**

 To increase reproducibility, I strongly recommend creating a GitHub page or a Supplementary file text containing all the commands with the selected parameters/options used across the bioinformatic pipeline. This will be very useful for the whole scientific community.

Are sufficient details of methods and materials provided to allow replication by others?

Partly

Is the rationale for creating the dataset(s) clearly described?

Yes

Are the datasets clearly presented in a useable and accessible format?

Yes

Are the protocols appropriate and is the work technically sound?

Yes

Reviewer Expertise:

Population genomics, Conservation genomics, Bioinformatics, Aquaculture

I confirm that I have read this submission and believe that I have an appropriate level of expertise to confirm that it is of an acceptable scientific standard.

## References

[1] : Global biotic interactions: An open infrastructure to share and analyze species-interaction datasets. *Ecological Informatics* .2014;24: 10.1016/j.ecoinf.2014.08.005 148-159 10.1016/j.ecoinf.2014.08.005
